# 2018 IEEE International Work Conference on Bioinspired Intelligence (IWOBI): Conference Report

**DOI:** 10.3390/biomimetics4010009

**Published:** 2019-01-25

**Authors:** Juan Luis Crespo-Mariño

**Affiliations:** Academic Area of Mechatronics Engineering and PaRMa Group, Tecnológico de Costa Rica, Cartago 30101, Costa Rica; jcrespo@tec.ac.cr

**Keywords:** IWOBI, bioinspired, transdisciplinary research, artificial intelligence, biocomputation, systems biology, high-performance computing

## Abstract

The International Work Conference on Bioinspired Intelligence (IWOBI) is an annual event that comprises both an international peer-reviewed scientific conference and a set of workshops and other activities in order to foster the research abilities and expertise of young researchers in the field of bioinspired intelligence. IWOBI 2018 has been characterized by a strong transdisciplinary component. The main conference themes were at the intersection between classical engineering disciplines and computer science, and the life and health sciences. This was motivated by the scientific environment that defines research that is being conducted in Costa Rica. Even though IWOBI is an international event, it was very important for the local organizing committee to focus on knowledge areas that were considered of special interest to Costa Rican researchers and to students looking to start their scientific careers. With such great expectations, IWOBI 2018 has been the first IWOBI conference in history to have parallel tracks. In addition to a regular track, a biocomputation and related techniques track was developed, as well as another one devoted to high-performance computing (HPC) systems applications for life and health sciences applications. Workshops were another important resource developed within IWOBI 2018. They were considered a very important tool in order to foster and train young researchers within the country and they are a very valuable chance to establish direct networking with elite researchers from different countries and research interests. IWOBI 2018 was the first IWOBI conference that implemented real and effective workshops. There were two workshops, one of them devoted to COPASI software and the other one focused on the use of the message passing interface (MPI) parallel programming library.

## 1. Introduction

The International Work Conference on Bioinspired Intelligence (IWOBI) is an international conference that was initially conceived as a framework for Spanish, Colombian, and Costa Rican research groups to communicate their results and to look for networking and coworking opportunities. This was the spirit that conducted the first two editions of the conference in 2012 and 2013 that were organized by a small group of professors belonging to a reduced number of institutions. However, in 2014, the situation changed when a large group of professors of several Costa Rican universities joined the project (which was initiated by Dr. Carlos Travieso at Universidad de Las Palmas de Gran Canaria, Spain). IWOBI 2014 was the first edition that obtained the support from the Institute of Electrical and Electronics Engineers (IEEE) and a larger number of contributions than former editions.

The expertise that the local Costa Rican team acquired was the motivation to develop a Costa Rica-own scientific conference that will maintain and even improve the multidisciplinary approach that was one of the key factors in the IWOBI conference. The first initiative was to develop a special session on bioinspired processing (BIP) within the 2016 Central America and Panama IEEE International Professional Convention (CONCAPAN). This event was considered highly successful and it was the beginning of a new international IEEE-supported conference when an application by the IWOBI steering committee was received in order to organize another edition of IWOBI 2018. This edition of the conference had the widest local organization team for a scientific conference in Costa Rica, as representatives from all public universities were participating in addition to the Costa Rica National Laboratory for High Performance Computing.

This organization effort allowed also that all five universities (Tecnológico de Costa Rica, Universidad de Costa Rica, Universidad Nacional de Costa Rica, Universidad Estatal a Distancia de Costa Rica and Universidad Técnica de Costa Rica) supported the event. A large impact on the Costa Rican research community was achieved. This was the reason for organizing two tracks in addition to the regular one. One of them was hosted by Prof. Rodrigo Mora at Universidad de Costa Rica, while for the other one, devoted to high-performance computing (HPC) applications in life and health sciences, Dr. Esteban Meneses and Dr. Francisco Siles served as the chairs. The general chair position of the IWOBI 2018 edition was assumed by Dr. Juan Luis Crespo at Tecnológico de Costa Rica (TEC).

The conference was held in San José (Costa Rica) for the workshops (16th and 17th July) and San Carlos (Costa Rica) TEC Campus for the conference (18th to 20th July). More than 50 papers were received from 14 countries all around the world. After an anonymous peer-review process with no less than three reviewers per paper, only 30 were accepted for oral presentation and publication. As was established before, the papers were presented in three different tracks. A brief summary of some of the most relevant contributions is presented in the following sections.

## 2. Regular Track

The regular track comprises the contributions that follow the main guidelines of the conference, that is, the intersection between technological and life and health sciences. In a more detailed manner, the papers presented within the regular track comprised, for example, the use of computer techniques for modelling components for medical devices (such as the paper by Zamora et al. [1]), or the development of subsystems for humanoid robots [2,3]. It is worth mentioning research results that, starting from the study of natural issues, present computer architectures that can be used in any kind of reinforcement learning application (see Becerra et al. [4]).

The regular track also included papers that showed results referring to the use of technological paradigms for solving nature-related problems. Applications for plant biology and conservation such as [5] and [6] are good examples.

Topics of interest for this track included, but were not limited to:

- optimization and metaheuristics
- biomathematics and biostatistics
- numerical methods and differential equations modeling
- pattern recognition and classification
- machine learning and computational intelligence techniques
- robotics
- signal processing and analysis
- computer vision
- intelligent networks
- bioinformatics
- computational anatomy
- natural sounds and speech recognition
- models of biological learning
- brain–machine interfaces
- speech and handwriting recognition

## 3. Biocomputation and Systems Biology Track

This is a brand-new track that included knowledge areas that were not covered in previous IWOBI conferences. It came from the previous expertise and background of the IWOBI 2018 organizing committee and their experience while organizing the BIP special session in 2016 (even their topics have been included in the IWOBI 2019 by its local organizing committee). There is a wide and relevant research expertise and background in Costa Rica, so it would be a relevant contribution to integrate the presentation of this research results within the conference. This will allow the establishment of international and multidisciplinary joint ventures among Costa Rican research labs and other research units all around the world.

Examples of some of the most relevant contributions within this track are the works of Brenes-Guillén et al. [7] on a systems biology approach to investigate control targets of intracellular calcium transients, Vargas et al. [8] on feature selection based on chromosomal regions alterations, Quiros-Fernandez et al. [9] on predicting cancer chemosensitivity based on intensity/distribution profiles of cells, or Campos-Sánchez and Sandoval-Carvajal [10] on the detection of endogenous retrovirus-derived transcripts in human testis.

Topics of interest for this track included, but were not limited to:

- dynamic models of metabolic, signaling, and gene expression networks
- improvements in genome assembly
- biological network reconstruction and analysis
- biomarker discovery and disease classification
- next-generation sequencing, copy number and gene expression analysis, proteomics, pharmacogenomics, epigenomics and other -omics, functional genomics
- molecular evolution and phylogeny
- protein folding and protein docking
- translational bioinformatics and immunoinformatics
- microbiomes applied to the field of conservation biology, disease, and health
- metagenomics of unusual environments
- applications of transcriptomics to study genome evolution and adaptation

## 4. High-Performance Computing for Life and Health Sciences

High-performance computing techniques are not only a more-than-needed resource for transdisciplinary research, since computing has evolved over time to be not only a tool but a research paradigm in itself, but also a tool that allows the optimization of physical research equipment and human resource use. Due to this fact, it was very important to establish a specialized track within the IWOBI 2018 devoted to the development of HPC paradigms and tools, and it also was a showroom for relevant research results of applications of HPC techniques to solve problems related to the life and health sciences.

The contributions that were presented within this track comprised papers exploring the parallelization of a denoising algorithm using OpenACC directives [11], the parallelization process of the deceived nonlocal means filter [12], a parallel implementation in a graphics processing unit (GPU) of the calculation of disparity maps for computer vision [13], and a comparative analysis of de Bruijn graph parallel genome assemblers [14].

Topics of interest for this track included, but were not limited to:

- parallel algorithms
- parallel programming techniques
- large-scale distributed systems
- high-performance applications and tools
- multicore architectures and accelerators
- grid and cloud computing and federations
- high-performance computing infrastructure and datacenters
- scientific and industrial computing
- big data and data management, and visualization

## 5. Keynote Speakers

Both at the IWOBI 2014 and 2018 conferences, the local organizing committee was very concerned about the high quality and international background and projection that the invited keynote speakers had to have. Keynote speaker masterclasses are not only a formal requirement but a very important tool in order to fulfill one of the most important interests when organizing a scientific meeting: to foster international networks and the development of highly transdisciplinary projects. The intention of the organizing team regarding keynote speakers was to cover the different research interests and international trends with three global top-level contributions. Here, we briefly summarize the contributions of few of our distinguished guest:

- Dr. Pedro Mendes is a professor at the University of Connecticut (Department of Cell Biology, and Center for Cell Analysis and Modeling, University of Connecticut Health Center, Center for Quantitative Medicine). He studied at the University of Lisbon and obtained his Ph.D. on the computer simulation of the dynamics of biochemical pathways from the University of Aberystwyth, Wales, UK, under the supervision of Prof. Douglas B. Kell. Prof. Mendes has published more than 120 manuscripts and has a total of 17,734 citations and a h-index of 49, according to Google Scholar. His background is in biochemistry and computer science. He has an interest and has conducted research in both areas and their joint application. His area of research is computational systems biology, where he has demonstrated leadership in biochemical modeling and simulation; he was the author of Gepasi, a popular and pioneering software package for modeling biochemical networks, which then became COPASI, now one of the top simulators for systems biology. He has co-led the development of two community metabolic reconstruction projects, for yeast and human metabolism. He was also involved in developing standards for systems biology (Systems Biology Markup Language (SBML) and Simulation Experiment Description Markup Language (SED-ML)). He carried out research in the area of functional genomics data analysis, particularly in metabolomics and reverse engineering methods. He is interested in modeling biological systems at the cellular and whole-body levels and has active projects on models of yeast metabolism, mammalian iron biochemistry, eukaryotic protein translation, and oxidative stress. He has mentored 19 postdoctoral researchers and 21 Ph.D. students (past and current).
- Prof. Alexander Gelbukh has a M.Sc. degree in Mathematics (with honors) by the M.V. State University Lomonosov of Moscow, Russia (1990), and has obtained a Doctor of Science (computer science) degree from the Institute of Scientific and Technical Information of All Russia (1995). He has been a professor, researcher and Head of the Laboratory of Natural Language and Text Processing of the Research Center in Computation (CIC) of the National Polytechnic Institute (IPN), Mexico, since 1997. He has been a visiting professor at the National University of Colombia, guest researcher at Waseda University, Japan, member of the Mexican Academy of Sciences since 2000, and a national researcher of Mexico (SNI) since 1998, currently with level 2, and he has been awarded with the Diploma in Research by the IPN. He is the founder and chair of the CICLing International Conference series. He has been honorary chair of several conferences. He is the founder editor-in-chief of the International Journal of Computational Linguistics and Applications (IJCLA) and editor-in-chief of the journal POLIBITS. Prof. Gelbukh is president of the Mexican Society of Artificial Intelligence and former president of the Mexican Association of Natural Language Processing. His main areas of scientific interest are computational linguistics and artificial intelligence. He is the author, co-author, or editor of more than 500 scientific publications; co-author of 4 books; and editor-in-chief or member of the editorial committee for more than 10 international journals. He has been president, honorary president, or president of the program committee of more than 20 international congresses. He has been the director of more than 20 doctoral theses. He has directed several research projects supported by the Government of Mexico (CONACyT) in the field of computational linguistics and information retrieval.
- Dr. Pascal Tyrrell is a data scientist—a combination of research methodologist, computer/database solutions architect, and innovator. He received his Ph.D. in medical sciences from the University of Toronto working in the area of pediatric rheumatology at SickKids (Toronto, Canada). Currently, he is the director of data science and an assistant professor with the Department of Medical Imaging at University of Toronto, where his aim is to understand, explore, and innovate where information and communications technology can be used effectively to encourage the translation of health research into action. Dr. Tyrrell is also appointed to the Department of Statistical Sciences where his research aims to establish useful guidelines for the quantity and quality of input data for machine learning in medical imaging research by investigating current methods, as well as developing new ones. Dr. Tyrrell has previous work experience in the computer and financial industries, and more recently, he has joined the medical device tech start-up company AceAge, Inc. as chief scientific officer.

## 6. Conclusions

The development of international scientific events in a transdisciplinary scenario is a key factor for the development of high-level research activities in developing countries like Costa Rica. Peer-reviewed and internationally supported events are not only a very valuable resource in order to showcase research results from local units but are also an opportunity to establish international relationships and learn about state-of-the-art developments as well as research guides and trends in a relaxed and informal setting. Furthermore, they are a very valuable resource to motivate research vocations in young undergraduate and graduate students. This is the reason why the organizing team of the 2014 and 2018 editions of IWOBI is working to have their own scientific conference on an annual basis, starting in 2019.
